# P-2241. Monocyte Distribution Width (MDW) as a Biomarker to Distinguish Septic Shock with Bacteremia from Cardiogenic Shock

**DOI:** 10.1093/ofid/ofae631.2394

**Published:** 2025-01-29

**Authors:** Kaylie Christine Goldner, Michael E DeWitt, Ryan C Maves

**Affiliations:** Wake Forest, Winston Salem, North Carolina; Atrium Wake Forest Baptist Health/ Wake Forest University School of Medicine, Winston-Salem, North Carolina; Wake Forest University School of Medicine, Winston-Salem, North Carolina

## Abstract

**Background:**

Shock is associated with high mortality in intensive care units with resultant urgency for rapid diagnosis. The monocyte distribution width (MDW) is a biomarker that is elevated in sepsis and septic shock and is readily obtained from routine leukocyte differential testing. It is unknown whether MDW is also elevated in other kinds of shock. The purpose of this study was to evaluate the potential of MDW to discriminate between cardiogenic and septic shock.

**Figure:**
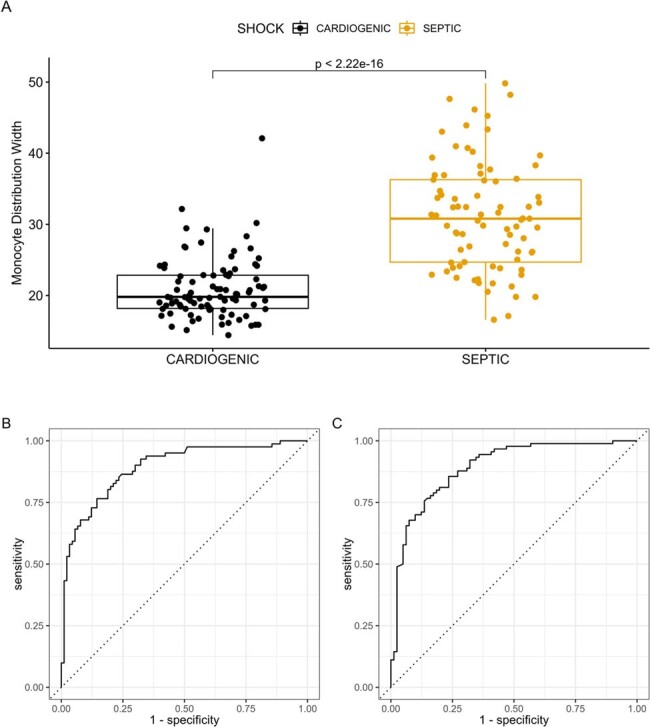

Boxplot of the MDW values by those with cardiogenic shock vs those with septic shock (A). The receiver operator curves for the prediction of septic shock (B) and cardiogenic shock (C) using MDW cut-off values, resulting in an AUC of 0.892.

**Methods:**

This study was a single-center retrospective analysis of adult patients admitted between 1/31/2023 and 12/27/2023 with either cardiogenic shock requiring inotropic or mechanical support or septic shock with bacteremia requiring vasopressor support derived from EHR database search for diagnosis codes. All patients required hemodynamic support and were excluded if any confounding immunosuppressive medications or conditions (e.g., advanced HIV infection, transplant, active malignancy on treatment) were present. Patients with cardiogenic shock were excluded if concomitant infection at the time of admission was identified. MDW was collected at the time of initial presentation.

**Figure:**
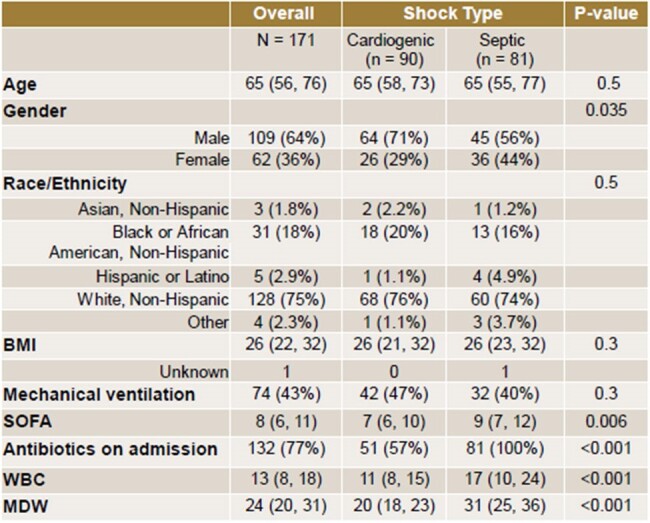

Study population demographics and results by shock category.

**Results:**

171 patients met inclusion criteria: 81 with septic shock and 90 with cardiogenic shock. 56.7% of patients with cardiogenic shock received empiric antibiotics on admission. The MDW was significantly higher in the septic shock group. Using estimated cut-off values of MDW > 21.53 for septic shock and MDW < 28.99 for cardiogenic shock each produced an AUC (area under the curve) of 0.892. Elevated SOFA score, elevated WBC, and female sex were associated with increased odds of septic shock. Adding the WBC count to MDW in the logistic regression for septic shock only minimally improved the AUC.

**Conclusion:**

MDW may be a useful tool in stratifying the risk of septic or cardiogenic shock. MDW above 28.99 supports a diagnosis of septic shock with bacteremia while MDW below 21.53 supports a diagnosis of non-septic shock; values from 21.53-28.99 are less predictive. A low MDW in patients with shock may exclude the need for empiric antibiotics and improve stewardship. Further work is needed to better characterize the performance of this assay in other populations and shock phenotypes.

**Disclosures:**

Ryan C. Maves, MD, AiCuris: Grant/Research Support|Biotest: Grant/Research Support|GeoVax: Grant/Research Support|Shionogi: Advisor/Consultant|Shionogi: Honoraria|Sound Pharmaceuticals: Grant/Research Support

